# Multi-omics integration reveals shared genetic architecture between metabolic markers and gray matter atrophy in Alzheimer’s Disease

**DOI:** 10.1016/j.tjpad.2025.100452

**Published:** 2026-01-01

**Authors:** Piaoran Wang, Xiangzheng Wu, Fengyu Sun, Hongchuan Zhang, Yurong Jiang, Qiuhui Wang, Hao Ding, Yujing Zhou, Feng Liu, Huaigui Liu

**Affiliations:** a Department of Radiology, Tianjin Key Laboratory of Functional Imaging & Tianjin Institute of Radiology, Tianjin Medical University General Hospital, Tianjin, China; b Department of Radiology, Yijishan Hospital of Wannan Medical College, Wuhu, Anhui, China; c Department of Radiology, The First Affiliated Hospital of Dalian Medical University, Dalian, Liaoning, China; d School of Medical Imaging, Tianjin Medical University, Tianjin, China; e Department of Radiology & Biomedical Imaging, Yale School of Medicine, New Haven, CT, USA

**Keywords:** Alzheimer’s disease, Metabolic markers, Gray matter volume, Gene expression, Genome-wide association studies

## Abstract

**Background:**

Alzheimer’s disease (AD) is a progressive neurodegenerative disorder characterized by widespread gray matter volume (GMV) reductions. Emerging evidence links glucose and lipid metabolic dysregulation to AD pathophysiology. However, the extent to which AD-related GMV alterations and metabolic traits share a common genetic basis remains poorly understood.

**Objectives:**

To explore the shared genetic architecture between GMV alterations in AD and metabolites related to glucose and lipid metabolism, aiming to provide biological insights into the prevention and treatment of AD.

**Design:**

This is a multimodal, cross-disciplinary study combining neuroimaging meta-analysis, transcriptome-neuroimaging association analysis, conjunctional false discovery rate (conjFDR) analysis, and functional enrichment analysis to identify the shared genetic architecture between AD-related brain structural alterations and metabolic traits.

**Setting:**

Public databases and European populations.

**Participants:**

The meta-analysis included 49 studies (1945 CE patients and 2598 controls). The largest genome-wide association study (GWAS) summary statistics were used for AD (N_case_ = 39,918; N_control_ =358,140), two glycemic traits—glucose (GLU, *N* = 459,772) and glycated hemoglobin (HbA1c, *N* = 146,864), and three lipid traits (*N* = 1320,016)—high-density lipoprotein cholesterol (HDL-C), low-density lipoprotein cholesterol (LDL-C), and triglycerides (TG).

**Measurements:**

We conducted a voxel-based morphometric meta-analysis of GMV in AD by systematically reviewing 49 neuroimaging studies, identified through a literature search in PubMed and Web of Science using a predefined search strategy. Building upon these neuroanatomical findings, we performed a transcriptome-neuroimaging association analysis using data from the Allen Human Brain Atlas to identify genes spatially correlated with GMV alterations. To further explore the shared genetic architecture, we integrated GWAS summary statistics for AD and five metabolic markers using conjFDR analysis. Finally, functional enrichment analyses were performed to elucidate the biological relevance of the identified genes through this integrative framework.

**Results:**

Consistent GMV reductions in AD were observed in the bilateral middle temporal gyrus, right superior temporal gyrus, and other key subcortical regions. The conjFDR analysis identified 20, 17, 78, 87, and 82 genes shared between AD-related GMV reductions and GLU, HbA1c, HDL-C, LDL-C, and TG, respectively. Notably, 6 genes were shared across all five metabolic markers. Enrichment analysis implicated these genes in biological processes related to Aβ aggregation and phosphatidylinositol metabolism.

**Conclusions:**

This study reveals a convergent genetic architecture underlying AD-related GMV atrophy and metabolic dysfunction. These findings may offer novel insights into the molecular interplay between systemic metabolism and neurodegeneration in AD and highlight potential targets for therapeutic strategies.

## Highlights

• **Multi-omics Integration:** Integrated voxel-based morphometry (VBM) meta-analysis, transcriptome-neuroimaging association analysis, and GWAS to identify pleiotropic genes.
• **Shared Genetic Architecture:** ConjFDR analysis revealed significant genetic overlap between AD-associated GMV atrophy and five metabolic markers, identifying 20–87 shared genes per metabolic trait.
• **Molecular Mechanisms:** Functional enrichment analysis elucidated the molecular interplay between metabolic dysregulation and neurodegenerative pathology, thereby identifying potential genetic targets for developing metabolism-focused targeted therapies for AD.

## 1 Introduction

Alzheimer's disease (AD) is the most prevalent neurodegenerative disease, affecting more than 50 million people worldwide [1]. It is characterized by progressive cognitive decline and pathological hallmarks such as β-amyloid (Aβ) accumulation, tau neurofibrillary tangles, and progressive gray matter volume (GMV) atrophy [[2], [3], [4]]. Notably, GMV atrophy has been observed decades before the onset of cognitive symptoms, positioning them as potential early biomarkers for AD. Voxel-based morphometry (VBM) studies have identified GMV reductions in key brain regions such as the hippocampus and entorhinal cortex. However, significant heterogeneity exists across studies, likely attributable to sample size limitations and methodological differences [[5], [6], [7]]. Neuroimaging meta-analysis can mitigate these limitations by aggregating data across studies, increasing statistical power, and identifying reproducible patterns of brain atrophy [8]. Previous voxel-based meta-analyses of GMV alterations in AD have been conducted using the Effect Size Signed Differential Mapping (ES-SDM) approach [9]. In light of methodological advances and the availability of additional neuroimaging studies, this meta-analysis was performed using the updated Seed-based d Mapping with Permutation of Subject Images (SDM-PSI) software, incorporating a larger and more comprehensive dataset to provide a more robust characterization of GMV changes in AD. Nevertheless, the molecular mechanisms underlying these GMV alterations remain largely unclear.

Recent studies highlight the potential role of metabolic dysregulation, particularly in glucose and lipid metabolism, in the development and progression of AD. The latest Genome-wide association studies (GWAS) have identified AD-associated genetic variants enriched in lipid metabolism pathways, including phospholipid efflux, cholesterol transport, and protein-lipid interactions [10]. In addition, other studies have identified risk genes directly involved in lipid metabolism and AD. Mechanistically, high cholesterol levels enhance the activity of β- and γ-secretases, promoting the amyloidogenic pathway of amyloid precursor protein (APP) and increasing Aβ production, while cholesterol accumulation in the brain is associated with increased tau phosphorylation and aggregation [11]. Additionally, polyunsaturated fatty acids (PUFAs) are prone to lipid peroxidation, producing excessive reactive aldehydes that elevate oxidative stress [11]. Similarly, impaired glucose metabolism has been implicated in AD [12]. Clinical studies have further supported these mechanisms, showing that elevated fasting glucose and dyslipidemia are associated with increased AD risk [[13], [14], [15]]. These findings point toward a potentially shared genetic basis between metabolic traits and AD pathology.

However, conventional GWAS approaches have a limited ability to capture pleiotropic effects across phenotypes and cannot localize molecular pathways within spatially defined brain regions [16]. To overcome these limitations, transcriptome-neuroimaging integration has emerged as a powerful framework [17,18]. By coupling spatial gene expression data from the Allen Human Brain Atlas (AHBA) with neuroimaging-derived GMV maps, researchers can map molecular pathways underlying structural brain changes [19,20]. Although prior studies have used this strategy to uncover gene expression patterns in AD [21,22], few have systematically linked these spatial transcriptomic profiles to metabolic genetic risk.

**Fig. 1:**
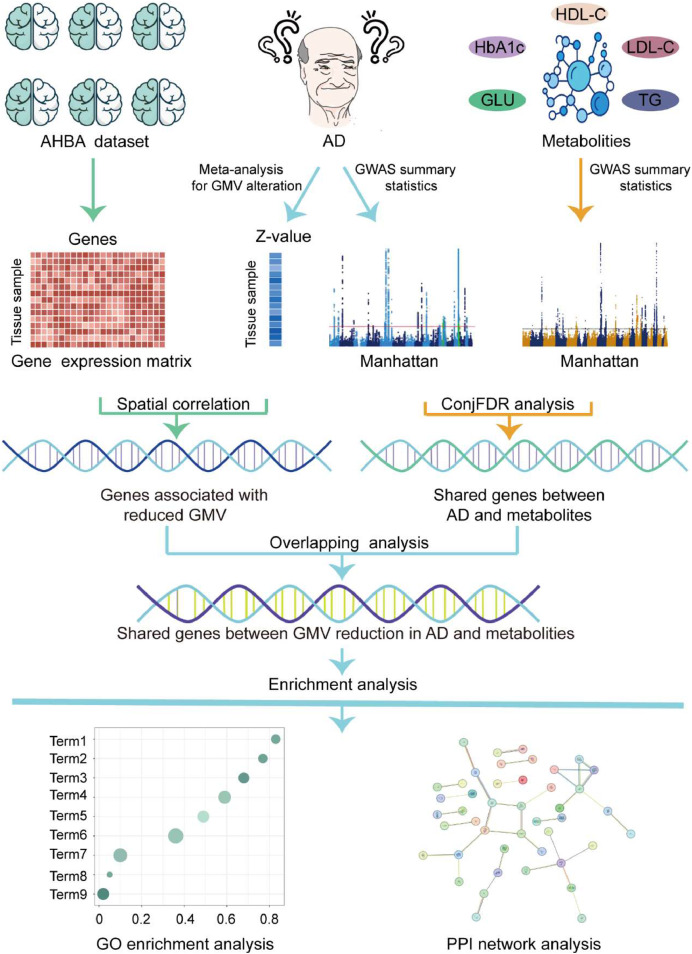
A schematic workflow of the multi-omics analytical framework. A coordinate-based neuroimaging-based meta-analysis is first conducted to identify GMV alterations associated with AD. Then, a transcriptome-neuroimaging association analysis is performed to identify genes associated with GMV alterations in AD. In parallel, the largest GWAS summary statistics of AD and five metabolic traits (GLU, HbA1c, TG, HDL-C, LDL-C) are analyzed using the conjFDR approach to detect pleiotropic genetic loci. An overlapping analysis between GMV alterations-associated genes and AD-metabolism shared genes is performed to identify genes potentially mediating the relationship between metabolic dysregulation and neurodegeneration. Finally, functional enrichment analysis is carried out to elucidate the biological functions of the intersecting genes.

**Abbreviations:** AD, Alzheimer's disease; AHBA, Allen human brain atlas; conjFDR, conjunctional false discovery rate; GLU, glucose; GMV, gray matter volume; HbA1c, Hemoglobin A1c; HC, healthy controls; HDL-C, High-Density Lipoprotein Cholesterol; LDL-C, Low-Density Lipoprotein Cholesterol; PPI, protein-protein interaction; TG, triglycerides.

To address this gap, we propose a multi-omics approach to unravel the shared genetic architecture underlying GMV alterations and metabolic dysregulation in AD. We first perform a neuroimaging meta-analysis to establish reproducible GMV alterations in AD. Next, we conduct transcriptome-neuroimaging association analysis to identify genes spatially associated with AD-specific GMV alterations [23,24]. In parallel, we apply conjunctional false discovery rate (conjFDR) analysis to integrate the largest available GWAS summary statistics for AD and five metabolic traits [10,[25], [26], [27]], identifying pleiotropic loci jointly shared between them. Finally, intersecting the gene sets derived from these two pipelines allows us to identify candidate genes that may link metabolic dysfunction to neurodegeneration. Functional enrichment analysis is then used to elucidate the biological pathways involved. This integrative study offers novel insights into the metabolic convergence of metabolic and neuroanatomical abnormalities in AD and may help identify genetic targets for therapeutic development. An overview of the analytical framework is presented in Fig. 1.

## 2 Materials and methods

### 2.1 Search strategy and selection criteria

A comprehensive literature search was conducted in the PubMed and Web of Science databases to identify relevant studies published before September 2024. The following keywords were used: ("Alzheimer's disease" OR "AD") AND ("voxel-based morphometry" OR "VBM" OR "voxel-based" OR "voxel-wise") AND ("gray matter" OR "grey matter" OR "cortex"). Additionally, the reference lists of the included studies were reviewed to identify other related studies.

**Fig. 2:**
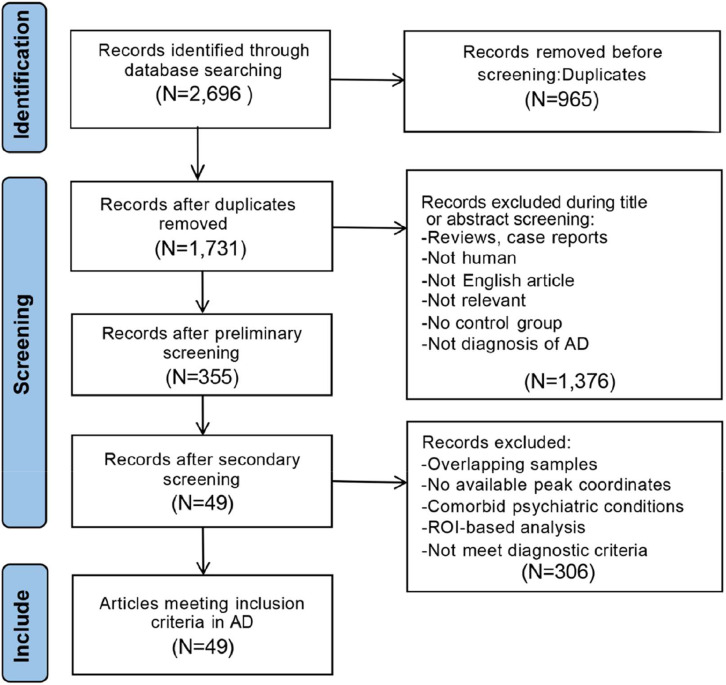
Flowchart of the literature search and selection criteria for the studies included in the meta-analysis. The process followed PRISMA guidelines, detailing the number of studies identified, screened, excluded, and finally included based on predefined inclusion and exclusion criteria.

**Abbreviations:** AD, Alzheimer’s disease; N, number; ROI, region of interest.

Studies were included if they met the following criteria: [1] published in peer-reviewed English-language journals; [2] included patients diagnosed with AD based on DSM-IV/DSM-IV-TR, NINCDS-ADRDA, or NIA-AA; [3] reported VBM comparisons (GMV) between patients with AD and HC; [4] provided peak coordinates of GMV alterations in Montreal Neurological Institute (MNI) or Talairach space, or reported null findings; [5] used statistical thresholds corrected for voxel-based multiple comparisons, or if uncorrected, reported spatial extent thresholds. Exclusion criteria included: [1] reviews, case reports, or similar studies; [2] insufficient sample size, with fewer than 9 participants in each group; [3] patients diagnosed with other diseases; [4] studies restricted to specific regions of interest (ROI). Our meta-analysis followed the Preferred Reporting Items for Systematic Reviews and Meta-Analysis (PRISMA) guidelines [28] with the detailed study selection process summarized in Fig. 2.

Two authors independently conducted literature screening and data extraction. The following variables were collected from each included study: sample size, demographic data such as age, gender distribution, and disease duration, as well as the software used, magnetic field strength, applied thresholds, and statistical values (e.g., *t*-values). Detailed study characteristics are summarized in **Table S1**.

### 2.2 Voxel-based neuroimaging meta-analysis

A voxel-based meta-analysis was conducted using the SDM-PSI software (version 6.22, available at https://www.sdmproject.com/) to explore GMV differences between AD patients and control groups. The analytical steps included multiple imputation of study images, imputation of subject images, group analysis of subject images for each study and each imputation, meta-analysis of study images using a random-effects model, and integration of the meta-analysis results using Rubin's rules [8]. To control the family-wise error (FWE) rate, we finally applied a subject-based nonparametric permutation test (1000 permutations) combined with threshold-free cluster enhancement (TFCE), with the significance threshold set at TFCE-FWE *P-*value < 0.05 [29]. Finally, heterogeneity was assessed using Cochran’s *Q* test and *I*² statistics to investigate potential variations in the findings, and Egger’s test was also applied to evaluate the potential for publication bias in the significant results [30].

To ensure the reliability of the results, meta-regression analysis was conducted to explore the potential impact of demographic variables such as mean age and the proportion of male patients on the results. Subgroup analysis was also performed to determine whether there were differences in the results across studies with various clinical and methodological characteristics. Specifically, we conducted subgroup meta-analyses on the following: [1] MRI with a 3.0T field strength, 27 studies; [2] smoothed with an 8 mm FWHM kernel, 34 studies. Due to the limited number of datasets available, no additional subgroup analyses were performed. The significance level was also set at TFCE-FEW *P-*value < 0.05. Detailed information can be found in the **Supplementary Methods**.

### 2.3 Transcriptome-neuroimaging association analysis

Gene expression data were obtained from the AHBA database, which provides comprehensive transcriptional profiles from 3702 brain samples across six different donors, covering 20,737 genes and utilizing 58,692 probes [31]. The demographic detail of each donor is displayed in **Table S2**. Among the six donors, two provided gene expression data for both hemispheres, while the remaining four contributed data exclusively from the left hemisphere. The gene expression data were preprocessed using the abagen toolbox, following the recommended pipeline [32]. First, microarray probes were reannotated to genes based on annotation information. Next, probes with expression levels below the background signal in more than 50 % of the samples were excluded. Then, the probe with the highest differential stability was selected to represent each specific gene. Subsequently, to minimize donor-specific variations in gene expression, the scaled robust sigmoid (SRS) method was applied to normalize expression values across genes for each tissue sample. Finally, additional normalization was performed within structural categories (e.g., cortex, subcortex/brainstem, and cerebellum) to account for significant differences in gene expression patterns across various brain structures. After processing, a sample-level gene expression matrix (1295 sample × 1,5633 genes) was generated for subsequent analysis.

We employed the PLS regression method [33,34] to investigate the correlations between the expression levels of 15,633 genes and alterations in GMV. In the PLS regression analysis, the predictor variable was the *z*-score normalized gene expression matrix consisting of 1295 regions and 15,633 genes, while the response variable was the *z*-score normalized case-control *t*-values of GMV. Subsequently, we calculated the variance explained by each PLS component between the predictor and response variables and arranged them in descending order.

We selected PLS components explaining more than 20 % of the variance for further analysis, as higher explained variance indicates a greater contribution to capturing the relationship between gene expression and imaging features [35]. To assess whether the variance explained by these components exceeded random expectations, we used the BrainSMASH tool (https://github.com/murraylab/brainsmash) to generate 1000 surrogate brain maps preserving the spatial autocorrelation of the original neuroimaging data, which is critical to avoid inflated false-positive results due to similarities among neighboring brain regions. We performed 1000 permutation tests using these surrogate maps to construct a null distribution of explained variance. The significance of each PLS component was determined by comparing its observed explained variance to this null distribution, with components showing *P*_perm_ < 0.05 considered significant. Furthermore, to estimate the contribution of each gene to the selected PLS component, we performed 1000 bootstrap resamples [36] and ranked gene contributions based on their normalized weight (*z*-score, defined as the weight divided by its standard error). Finally, to ensure the reliability of the results, we applied Bonferroni correction and identified genes with a significance level of *P-*value < 0.05 (either positive or negative *z*-scores). These significant genes were retained for subsequent analysis.

### 2.4 Shared genes between AD and metabolic markers revealed by conjFDR

#### 2.4.1 Selection of metabolic markers

Previous studies have demonstrated the strong links between glucose metabolism, lipid metabolism, and the pathophysiology of AD [11,12]. Given the broad spectrum of circulating metabolic markers, we selected five well-characterized and clinically relevant biomarkers to represent these two pathways. Specifically, associated with glucose and lipid metabolism, a selection of representative markers was made. For glucose metabolism, glucose (GLU) and glycated hemoglobin (HbA1c) were chosen to reflect glucose metabolism, while high-density lipoprotein cholesterol (HDL-C), low-density lipoprotein cholesterol (LDL-C), and triglycerides (TG) were selected as indicators of lipid metabolism.

#### 2.4.2 ConjFDR analysis to identify pleiotropic genes

To explore the shared genetic mechanisms between AD and metabolic markers, the largest publicly available GWAS summary statistics were utilized. For AD, GWAS data were obtained from the Psychiatric Genomics Consortium (PGC, https://pgc.unc.edu/for-researchers/download-results/). To avoid potential sample overlap, participants from the UK Biobank were excluded, and due to data access restrictions, data from 23andMe were also omitted. This resulted in a final sample size of 39,918 cases and 358,140 controls of European ancestry [10].

For metabolic traits, GWAS summary data for lipid metabolic markers (HDL-C, LDL-C, TG) were derived from the Global Lipids Genetics Consortium (http://csg.sph.umich.edu/willer/public/glgc-lipids2021), encompassing 1320,016 individuals of European ancestry [25]. The GWAS summary statistics for glucose metabolic markers (GLU and HbA1c) were obtained from the MAGIC consortium (https://magicinvestigators.org/), with GLU data including 459,772 individuals of European ancestry and HbA1c data including 146,864 individuals of European ancestry [26,27]. Using these GWAS summary statistics, we performed conjFDR analysis to identify shared genetic variants between AD and these metabolic markers [37]. The conjFDR analysis, an extension of the conditional FDR (condFDR) method based on an empirical Bayesian statistical framework, aims to identify shared variants by leveraging single-nucleotide polymorphism (SNP) associations between two phenotypes [38]. In condFDR analysis, test statistics are re-ranked, and the associations of variants with the primary phenotype are adjusted based on their associations with the secondary phenotype. By reversing the roles of the primary and secondary phenotypes, the inverse condFDR value is obtained, with the conjFDR value defined as the larger of the two mutual condFDR values. Consistent with previous studies, variants with conjFDR < 0.05 were considered statistically significant [39]. Enrichment was visualized using stratified quantile-quantile (Q-Q) plots, where SNP *P-*value distributions for the primary phenotype were conditioned on *P*-value thresholds for the secondary phenotype (e.g., *P-*value < 0.100, 0.010, 0.001). Leftward deviation from the expected line indicates that the secondary phenotype enhances the association significance of the primary phenotype. To reduce potential biases caused by linkage disequilibrium (LD) in complex regions, we excluded SNPs located in the extended major histocompatibility complex (MHC) region (chr6: 25,119,106–33,854,733), the 8p23.1 region (chr8: 7242,715–12,483,982) [40].

We used the FUMA platform to define independent genomic loci [41]. Specifically, SNPs (i.e., genetic variants) with conjFDR < 0.05 and LD *r*² < 0.6 were identified as independent significant SNPs, while those with *r*² < 0.1 were considered lead SNPs. Candidate SNPs were defined as SNPs with a conjFDR value of < 0.10 and an LD *r*^2^-value of > 0.60 with an independent significant SNP [42,43]. Loci within 250 kb were merged, with the minimum FDR value determining the final lead SNP. Subsequently, all candidate SNPs were functionally annotated using CADD scores [44], RegulomeDB scores [45], and chromatin state information [46,47]. To align the candidate SNPs with potential causal genes, we used positional mapping to map the candidate SNPs to genes. The genes mapped through this strategy were defined as the shared genes between AD and metabolic markers.

### 2.5 Shared gene identification

To investigate genetic convergence, we intersected the gene set AD-related GMV alterations (identified via transcriptome-neuroimaging association analysis) and the conjFDR-derived gene set shared between AD and each metabolic marker. This yielded five gene sets, corresponding to genes jointly implicated in AD-related GMV alterations and AD-metabolic marker pairs of GLU, HbA1c, HDL-C, LDL-C, and TG, respectively. Additionally, we derived the intersection across all five sets to investigate a core group of pleiotropic genes potentially underlying a common mechanism linking metabolic dysregulation and neurodegeneration in AD. To facilitate the visualization of gene overlaps, we created a Venn diagram.

### 2.6 Enrichment analysis

To explore the biological functions of the intersecting genes, we conducted a Gene Ontology (GO) enrichment analysis, which classifies genes into hierarchical categories across three domains: biological process, molecular function, and cellular component. Functional annotations based on the GO database were implemented in R, and multiple comparison corrections were performed using the false discovery rate (FDR) method (*P-*value < 0.05) to investigate the biological functions of the identified genes. In addition, a protein-protein interaction (PPI) network for the shared genes was constructed using the STRING database (version 12.0, https://string-db.org/) with a medium confidence threshold of 0.4 [48].

## 3 Results

### 3.1 Included studies and sample characteristics

As shown in Fig. 2, our search strategy identified a total of 2696 studies. After removing 965 duplicate studies using EndNote X9 and manual screening, 49 studies on AD-related GMV met the inclusion criteria for meta-analysis. These studies reported 55 datasets, comprising 1945 CE patients and 2598 healthy controls. The demographic, clinical characteristics, and imaging information of the subjects are summarized in **Table S1**.

### 3.2 Neuroimaging meta-analysis revealed GMV alterations in AD

**Fig. 3:**
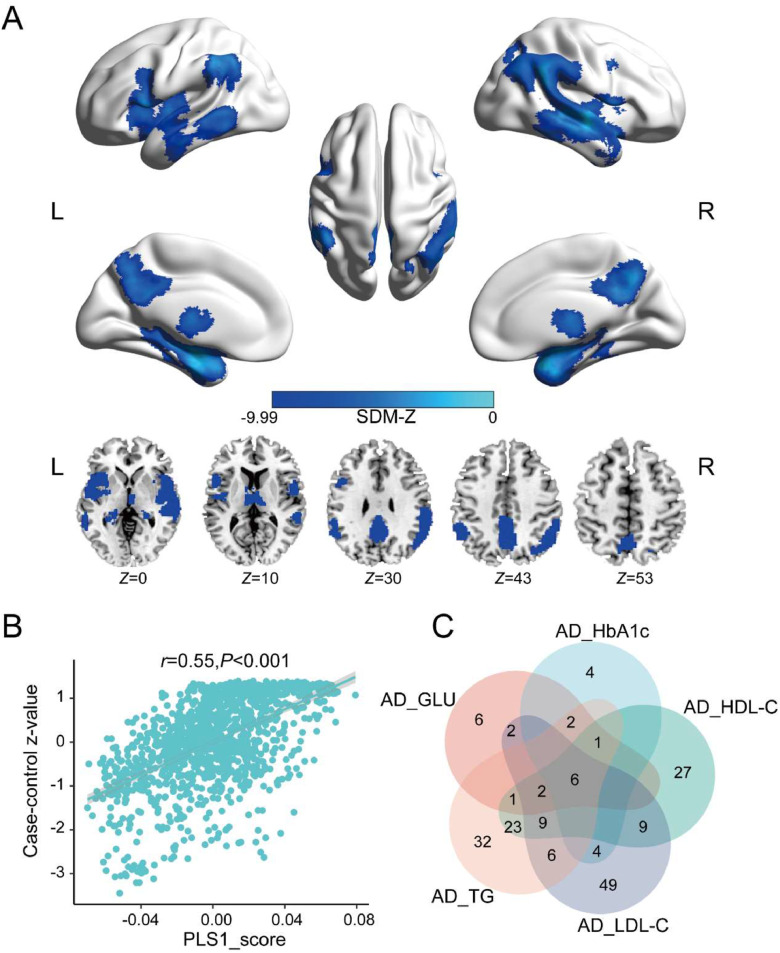
Regions of significantly decreased GMV in AD and gene expression analysis and overlapping gene analysis results. **A.** Brain regions showing significant GMV reductions in AD. The color bar represents the SDM-*Z* values. **B.** A scatterplot illustrating the PLS1 score (x-axis) and case-control *z*-map (y-axis) (Pearson's *r* = 0.5512, *P-*value <0.001). **C.** The Venn diagram illustrates the common genes shared across five gene sets, each derived from the overlap between genes associated with AD-related GMV alterations and those associated with one of five AD-related metabolic markers.

**Abbreviations:** GLU, glucose; HbA1c, Hemoglobin A1c; HDL-C, High-Density Lipoprotein Cholesterol; LDL-C, Low-Density Lipoprotein Cholesterol; L, left; PLS1, the first component of the partial least squares regression; R, right; SDM, seed-based d mapping; TG, triglycerides.

Neuroimaging meta-analysis identified consistent GMV reductions in AD patients compared to controls. Significant atrophy was observed in the bilateral middle temporal gyrus (MTG), right superior temporal gyrus (STG), supramarginal gyrus (SMG), right angular gyrus (AG), hippocampus (HPC), thalamus (Th), bilateral precuneus (PCUN), middle cingulate cortex (MCC), left inferior parietal lobule (IPL), posterior inferior temporal gyrus (PITG), and right middle frontal gyrus (MFG) (Fig. 3**A** and Table 1). No significant increases in GMV were observed. Subsequently, Cochran's *Q* test and *I*² statistics indicated no significant heterogeneity among studies in the reported results, and Egger's test indicated no evidence of publication bias in the significant clusters. Detailed results of the meta-regression and subgroup analysis are provided in the Supplementary Results.

**Table 1:** Results of meta-analysis between Alzheimer's disease patients and controls.

Brain region	SDM-Z	*P-*value	Peak MNI coordinates	Cluster size(voxels)	Heterogeneity test		Egger’s test
			X Y Z		Q (*P-*value)	*I*^2^ ( %)	*P-*value
AD<control groups							
MTG/STG_R/SMG_R/AG_R/HPC/Th	−9.99	∼0	24, 4, −28	20,558	49.61(0.64)	30.26	0.60
Bilateral PCUN/MCC	−9.19	∼0	2, −56, 38	2310	41.27(0.89)	11.20	0.37
IPL_L/SMG_L	−6.95	∼0	−58, −44, 40	833	45.86(0.78)	29.68	0.51
MTG/PITG	−5.41	0.007	−54, −52, −8	745	34.67(0.98)	3.69	0.58
MFG_R	−5.45	0.038	42, 6, 38	35	33.53(0.99)	15.65	0.48

**Abbreviations:** AG, Angular Gyrus; HPC, Hippocampus; IPL, Inferior Parietal Lobule; MNI, Montreal Neurological Institute; MTG, Middle Temporal Gyrus; MFG, Middle Frontal Gyrus; MCC, Middle Cingulate Cortex; PCUN, Precuneus; PITG, Posterior Inferior Temporal Gyrus; Q, Cochran’s Q statistic; SDM, seed-based d mapping; STG, Superior Temporal Gyrus; SMG, Supramarginal Gyrus; Th, Thalamus; L, Left; R, Right.

### 3.3 Meta-regression analysis and subgroup analysis

Subgroup analysis revealed significant heterogeneity only in the 3.0T field strength subgroup, specifically in the left hippocampus and parahippocampal gyrus (*P* < 0.05, *I*² = 77.39), where publication bias was also detected (Egger’s test, *P-*value< 0.04). No significant heterogeneity or bias was observed in other clusters (see **Table S3–4, Supplementary Figure 1**). The results of the meta-regression are summarized in **Table S5**, and the detailed results of the meta-regression and subgroup analysis are provided in the supplementary results.

### 3.4 Transcriptome-neuroimaging association analysis

Following gene expression preprocessing, we applied PLS regression to identify genes spatially associated with AD-related GMV alterations. The first PLS component (PLS1) accounted for over 20 % of the variance and was significantly positively correlated with the case-control difference *z*-map (*r* = 0.5512, *P-*value < 0.001, Fig. 3**B, Supplementary Figure 2**). A spatial autocorrelation-preserved permutation test further confirmed the robustness of these findings, showing that the variance explained by PLS1 exceeded that expected under the null distribution (permutation *P-*value = 0.0013). Based on the *z*-scores of each gene and ranked by the normalized weights of PLS1, a total of 5349 genes were identified as significantly contributing to PLS1 (*P-*value < 0.05, Bonferroni corrected) (**Table S6–7**).

### 3.5 Shared genes between AD and metabolic markers revealed by conjFDR

**Fig. 4:**
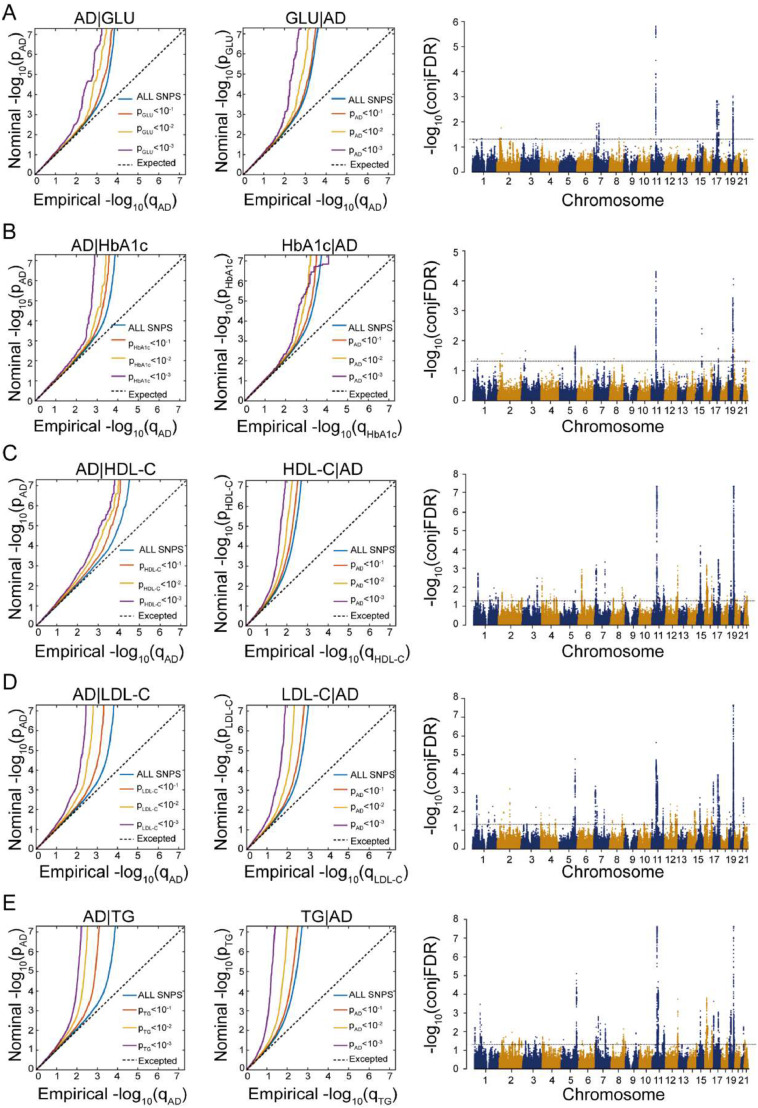
Results of conjFDR analysis between AD and five metabolic markers. **A.** Results of conjFDR analysis between AD and GLU. **B.** Results of conjFDR analysis between AD and HbA1c. **C.** Results of conjFDR analysis between AD and HDL-C. **D.** Results of conjFDR analysis between AD and LDL-C. **E.** Results of conjFDR analysis between AD and TG. Conditional Q-Q plot: Under the conditions of AD and five metabolic markers, the comparison of nominal and empirical -log_10_*P-*value is shown at levels of *P-*value < 0.100, *P-*value < 0.010, and *P-*value < 0.001. The blue line represents all SNPs, and the dashed line indicates the null hypothesis. Manhattan plot showing the -log_10_-transformed conjFDR values for each SNP on the y-axis and chromosomal positions along the x-axis. The dotted horizontal line represents the threshold for significant shared associations (conjFDR < 0.05).

**Abbreviations:** conjFDR, conjunctional false discovery rate; GLU, glucose; HbA1c, Hemoglobin A1c; HDL-C, High-Density Lipoprotein Cholesterol; LDL-C, Low-Density Lipoprotein Cholesterol; TG, triglycerides.

The conditional Q-Q plots showed significant genetic enrichment between AD and all five metabolic markers, indicating pleiotropic effects. Under the condition of conjFDR < 0.05, we identified independent genomic loci and corresponding genes jointly associated with AD and five metabolic markers: GLU (15 loci, 102 genes), HbA1c (17 loci, 78 genes), HDL-C (84 loci, 354 genes), LDL-C (86 loci, 386 genes), and TG (72 loci, 338 genes) (see Fig. 4**A-E**). Details of these independent genomic loci and functional annotation results are provided in **Table S8–22**. We intersected these genes with the 5349 significant genes associated with AD-related GMV alterations, identifying 20 genes shared with GLU, 17 with HbA1c, 78 with HDL-C, 87 with LDL-C, and 82 with TG (**Table S23**). The further intersection of shared genes related to the five metabolic markers and AD-related GMV alterations yielded six core genes: *KBTBD4, FNBP4, MYBPC3, SLC39A13, PTPMT1*, and *NUP160* (**Table S23**). To visualize these results, we created a Venn diagram (Fig. 3**C**).

### 3.6 Enrichment analysis

**Fig. 5:**
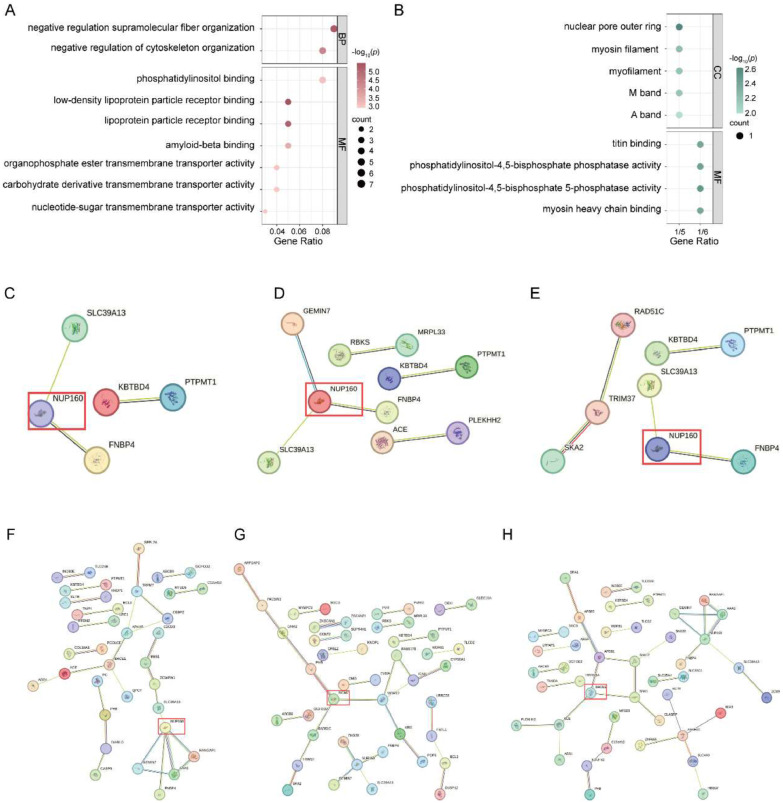
Enrichment analysis of identified overlapping genes. **A.** Enriched terms of overlapping genes between AD-related GMV alterations and TG. **B.** The top 9 enriched terms of overlapping genes are shared by AD-related GMV alterations and all five metabolic markers. The x-axis represents the gene ratio (intersection size/query size) of each term, and the y-axis shows the GO pathway, and the shade of color indicates the -log_10_(*P*) with the FDR corrected *P-*value. **C.** PPI network of the six overlapping genes shared between AD-related GMV alterations and the five metabolic markers. **d-H.** PPI network of overlapping genes between AD-related GMV alterations and GLU, HbA1c, HDL-C, LDL-C, and TG, respectively.

**Abbreviations:** BP, biological processes; CC, cellular components; FDR, False discovery rate; MF, Molecular Function; PPI, protein-protein interaction

We performed GO enrichment analysis using R to explore the biological functions of the shared genes identified. The results showed that the shared genes between AD-related GMV alterations and TG exhibited significant enrichment in the GO analysis. These genes were associated with biological processes related to cellular structure, lipid metabolism, receptor binding, as well as membrane and transport functions. The enrichment analysis of genes shared between AD-related gray matter atrophy and each of the other four metabolic traits did not yield significant results. The six genes shared between AD-related GMV alterations and all the five metabolic markers were enriched in terms related to cellular structure and muscle function, phospholipid metabolism, and signal transduction-related functions (Fig. 5**A-B, Table S24–25**).

The PPI network analysis revealed that the networks constructed by genes overlapping between AD-related GMV alterations and each metabolite were significantly higher than expected: genes associated with GLU formed a network with 6 edges (expected 1, *P-*value < 0.001); HbA1c, 5 edges (expected 0, *P-*value < 4.1e-05); HDL-C, 29 edges (expected 14, *P-*value < 0.001); LDL-C, 32 edges (expected 18, *P-*value < 0.002); and TG, 35 edges (expected 12, *P-*value < 3.44e-08). Additionally, the six genes shared between AD-related GMV alterations and all the five metabolic markers constructed a network with 3 edges (expected 0, *P-*value < 1.44e-05). Through betweenness centrality analysis of node scores in these networks, we found that the protein encoded by *NUP160* serves as a key node in the PPI networks of AD with GLU, HbA1c, HDL-C, and the shared network of AD with the five metabolic markers. The protein encoded by *BACE1* is crucial in the AD-TG network, while the protein encoded by *MCM7* plays a key role in the AD-LDL-C network (Fig. 5**C—H**).

## 4 Discussion

This study is the first to systematically explore the shared genetic architecture underlying GMV alterations in AD and glucose- and lipid-related metabolic markers. By integrating neuroimaging meta-analysis and transcriptome-neuroimaging association analysis, we identified genes spatially associated with AD-related GMV reduction. Leveraging large-scale GWAS summary statistics and applying the conjFDR approach, we further pinpointed pleiotropic genes associated with AD and five key metabolic markers. Our cross-analysis revealed substantial genetic overlap, identifying 20 genes shared between AD-related GMV alterations and GLU, 17 with HbA1c, 78 with HDL-C, 87 with LDL-C, and 82 with TG. Notably, six genes were consistently associated with AD-related GMV alterations and all five metabolic markers, highlighting potential convergent pathways linking metabolic dysregulation and neurodegeneration in AD.

Neuroimaging meta-analysis identified that GMV alterations in AD patients are distributed across multiple brain regions, and associated with various cognitive impairments. Notably, subgroup analysis focusing on studies conducted at 3.0T field strength revealed significant heterogeneity and publication bias in the left HPC and PHG. This heterogeneity may partly reflect the technical advantages of high-field MRI. Compared to 1.5T scanners, 3.0T systems offer higher spatial resolution and signal-to-noise ratio, enabling more sensitive detection of subtle structural changes [49], which could contribute to greater variability across studies. The observed publication bias also indicates a tendency for positive findings to be preferentially published, particularly in studies with smaller sample sizes, reflecting a broader issue of "significance bias" in neuroimaging research. Such bias may in part be driven by underlying heterogeneity among included studies [50]. These findings underscore the need for standardized imaging protocols, larger sample sizes, and improved reporting of non-significant results to enhance the reproducibility and reliability of future studies.

Specifically, these affected brain regions can be broadly categorized into three groups: regions supporting language and memory, regions associated with emotion and social cognition, and regions involved in executive function and motor control. The MTG and STG play important roles in language processing and semantic memory [51,52]. Studies have shown that AD patients experience a decline in language comprehension and semantic memory deterioration, aligning with our findings of significant GMV reduction in these regions [53]. Within the memory network, the HPC serves as a critical hub for the formation and consolidation of episodic memories [54]. The observed GMV reduction in the HPC may account for the early-onset memory deficits frequently reported in patients with AD. Furthermore, the AG is implicated in episodic simulation and memory [55], while the PCUN plays a key role in working memory and self-referential thought, both of which are frequently impaired in AD [56]. In addition to language and memory dysfunctions, AD is also characterized by deficits in emotional and social cognition. The SMG is essential for emotion recognition [57], whereas the MCC is involved in emotion regulation and pain perception [58]. In parallel, the thalamus, due to its unique anatomical position - between the brainstem and the cerebral cortex - hints its function as an information relay station and a hub for communication [59]. Together, these regions are involved in the integration of emotional and sensory information. GMV reductions in these areas may impair emotional perception and pain processing in patients with AD, potentially contributing to the emotional blunting and social interaction difficulties commonly observed in this population. Importantly, GMV reductions in the IPL and MFG may underlie deficits in motor execution and decision-making of AD patients [60,61], resulting in a decline in daily living skills and difficulty completing complex tasks. In summary, our findings underscore the widespread structural degeneration across multiple functional systems in AD. Further investigation into these affected brain areas may yield deeper insights into the neurobiological mechanisms of AD and inform the development of targeted interventions aimed at preserving cognitive function.

AD is increasingly recognized as a multifactorial disorder, with metabolic dysfunction emerging as a central pathogenic component [11,12]. Our conjFDR-based analysis revealed hundreds of pleiotropic genetic loci shared between AD and each metabolic marker, including 15 and 17 loci for GLU and HbA1c, and 72–86 loci for TG, HDL-C, and LDL-C. When intersected with transcriptome-inferred GMV-related genes, we refined these to a smaller set of biologically meaningful candidates. Notably, six genes were consistently shared across all five metabolic traits, suggesting convergent molecular mechanisms linking systemic metabolism and brain structural changes in AD. Among these, *SLC39A13* encodes a zinc ion transporter, and zinc homeostasis plays a crucial role in the pathological mechanisms of AD. Zinc ions are known to promote the aggregation of Aβ and enhance its neurotoxicity [62,63], while dysregulated zinc metabolism may affect synaptic formation and synaptic plasticity, thereby potentially exacerbating neurodegenerative alterations in AD [64]. Notably, previous studies have also reported altered zinc regulatory protein levels in AD patients, supporting the relevance of *SLC39A13* in disease progression [65]. Another key gene, *PTPMT1*, encodes a mitochondrial protein tyrosine phosphatase essential for maintaining mitochondrial flexibility and energy metabolism [66]. Mitochondrial dysfunction is a hallmark of AD [67,68], and recent finding suggests that impaired mitophagy contributes to synaptic dysfunction and cognitive deficits by increasing oxidative damage and cellular energy deficits, which in turn promote the accumulation of Aβ and Tau [69]. These findings imply that *PTPMT1* may influence the progression of AD by regulating mitochondrial function and affecting neuronal energy metabolism. GO enrichment analysis of the shared genes further reinforced these findings. For TG-related genes, Aβ binding emerged as a significantly enriched term. Given the role of Aβ plaque formation in AD pathophysiology, genes involved in Aβ binding may influence aggregation, clearance, or transport [70,71]. Notably, TG has been shown to affect Aβ transport, and dysregulation in TG may affect Aβ clearance, accelerating its accumulation and toxicity [72,73]. In addition, GO enrichment analysis of the six convergent revealed significant enrichment for phosphatidylinositol metabolism, including phosphatidylinositol (PIP2) phosphatase activity, suggesting a link to cell signaling pathways that may modulate neuronal integrity [74].

Our PPI network analysis further demonstrated that *NUP160* exhibits a central role in the PPI network, suggesting its potential involvement in the pathological mechanisms of AD-related GMV alterations and metabolic disorders. *NUP160* is an essential component of the nuclear pore complex (NPC), which plays a crucial role in nucleocytoplasmic transport. Previous studies have shown that dysfunction of the NPC is associated with various neurodegenerative diseases, such as AD, amyotrophic lateral sclerosis (ALS), and Huntington's disease (HD) [75]. Numerous studies have shown that the NPC can regulate gene expression [76,77]. Furthermore, research has found that NPC dysfunction can lead to tau-induced neurotoxicity in AD and tauopathies [78]. Although direct evidence on the role of *NUP160* in AD is currently limited, its central role in nucleocytoplasmic transport and gene regulation suggests that it may contribute to GMV reduction and metabolic dysregulation in AD. Future studies are warranted to determine whether modulation of *NUP160* expression or function influences neurodegenerative or metabolic processes in AD. Such investigations could provide novel insights into the mechanistic role of *NUP160* and may support its potential as a diagnostic biomarker or therapeutic target.

Our study reveals a shared genetic architecture linking AD-related gray matter atrophy with metabolic traits related to glucose and lipid metabolism. A recent multi-omics investigation that combined 16S rDNA amplicon sequencing, untargeted metabolomics, and multimodal MRI to examine gut microbiota, fecal metabolome, neuroimaging phenotypes, and cognitive variables across AD patients, individuals with cognitive impairment, and healthy controls. Through correlation and mediation analyses, the study identified two potential biological pathways: [1] a sequential pathway from gut microbiota to metabolites, then to neuroimaging features, and ultimately to cognition;, and [2] a more direct pathway from gut microbiota to metabolites and then to cognitive outcomes [79]. These findings provide valuable complementary insights to our work and suggest future research could benefit from incorporating broader metabolic features, exploring mediation mechanisms linking systemic metabolism, neuroimaging, and cognition or clinical symptoms, and identifying key regulatory genes that may serve as promising therapeutic targets to enhance clinical translational potential.

Several limitations should be noted. First, the meta-analysis relying on cross-sectional data cannot establish causal relationships between GMV alterations and AD. Second, although two independent researchers conducted the literature search, the results may still be subject to subjective bias, which could affect the inclusion of relevant studies and impact the meta-analysis results. Third, the gene expression and imaging data used for the transcriptome-neuroimaging association analysis were not from the same subjects, and further research using multi-omics datasets from the same individuals is needed to improve the reliability and biological interpretability of the findings.

## 5 Conclusion

In summary, our study reveals the shared genetic landscape linking AD-related GMV alterations with classic markers of glucose and lipid metabolism, uncovering six convergent genes (e.g., *SLC39A13, PTPMT1*) potentially linking neurodegeneration with glucose and lipid dysregulation. By integrating voxel-based meta-analysis, spatial transcriptomics, and GWAS-based pleiotropy analysis, we identified molecular pathways—including Aβ binding and phosphatidylinositol metabolism—that may underlie these associations. These findings not only deepen our understanding of the metabolic mechanisms driving AD-related GMV alterations but also highlight potential therapeutic avenues, such as targeting metabolic pathways, modulating gene expression, or developing biomarkers for early intervention and disease monitoring.

## Data availability

All GWAS summary data used in our study are publicly available. The AD GWAS summary data were obtained from the Psychiatric Genomics Consortium (PGC: https://pgc.unc.edu/for-researchers/download-results/); the lipid metabolism GWAS summary data were obtained from the Global Lipids Genetics Consortium (http://csg.sph.umich.edu/willer/public/glgc-lipids2021); and the GWAS summary data for GLU and HbA1c were obtained from the MAGIC consortium (https://magicinvestigators.org/).

## Funding

This work was supported by Tianjin Major Special Project on Public Health Science and Technology (24ZXGQSY00040), and the Tianjin Key Medical Discipline (Specialty) Construction Project (TJYXZDXK-008C).

## Ethical standards

This study utilized de-identified, publicly available secondary data and therefore did not require informed consent and ethical approval.

## Declaration of generative AI and AI-assisted technologies in the writing process

No artificial intelligence tools were used in the conceptualization, data analysis, or drafting of this manuscript. ChatGPT was employed solely for minor language editing after the manuscript was completed, contributing to approximately 5 % of the final content refinement.

## CRediT authorship contribution statement

**Piaoran Wang:** Writing – original draft, Visualization, Validation, Software, Methodology. **Xiangzheng Wu:** Visualization, Validation, Software, Methodology. **Fengyu Sun:** Visualization, Software, Methodology. **Hongchuan Zhang:** Visualization, Software, Methodology. **Yurong Jiang:** Visualization, Software, Methodology. **Qiuhui Wang:** Software, Methodology. **Hao Ding:** Software, Methodology. **Yujing Zhou:** Writing – review & editing, Project administration, Conceptualization. **Feng Liu:** Writing – review & editing, Project administration, Conceptualization. **Huaigui Liu:** Writing – review & editing, Validation, Supervision, Software, Project administration, Methodology, Funding acquisition, Conceptualization.

## Declaration of competing interest

The authors declare that they have no known competing financial interests or personal relationships that could have appeared to influence the work reported in this paper.

## Appendix Supplementary materials

Image, application 1

Image, application 2
