# Supplementary material for: Multi-omics integration reveals shared genetic architecture between metabolic markers and gray matter atrophy in Alzheimer’s Disease
Source: J Prev Alzheimers Dis. 2026 Jan 1;13(2):100452. doi: 10.1016/j.tjpad.2025.100452 (PMC12869038; doi:10.1016/j.tjpad.2025.100452)
Supplement: Supplementary file 1 [file mmc1.docx]

**Supplementary Materials for**

**Multi-omics Integration Reveals Shared Genetic Architecture Between Metabolic Markers and Gray Matter Atrophy in Alzheimer’s Disease**

Piaoran Wang^1#^, Xiangzheng Wu^1#^, Fengyu Sun^1#^, Hongchuan Zhang^2#^, Yurong Jiang^1^, Qiuhui Wang^1^, Hao Ding^1,4^, Yujing Zhou^3^*, Feng Liu^1^*, Huaigui Liu^1,5^*

^1^Department of Radiology, Tianjin Key Laboratory of Functional Imaging & Tianjin Institute of Radiology, Tianjin Medical University General Hospital, Tianjin, China

^2^Department of Radiology, Yijishan Hospital of Wannan Medical College, Wuhu, Anhui, China

^3^Department of Radiology, The First Affiliated Hospital of Dalian Medical University, Dalian, Liaoning, China

^4^School of Medical Imaging, Tianjin Medical University, Tianjin, China

^5^Department of Radiology & Biomedical Imaging, Yale School of Medicine, New Haven, CT, USA.

^#^These authors contributed equally to this work.

***Correspondence to:** Huaigui Liu, Email: liuhuaigui@tmu.edu.cn; Feng Liu, fengliu@tmu.edu.cn; Yujing Zhou, yjzhou0613@163.com.

**Supplementary Methods**

**Additional analysis**

To further examine the robustness of the meta-analysis findings and identify potential moderating factors, we conducted the following exploratory analysis. First, meta-regression analyses were carried out to explore the impact of the proportion of male participants and age on GMV alterations in AD. Secondly, subgroup meta-analyses were performed to evaluate the consistency of the findings and to identify potential factors that might influence the primary outcomes, including only those studies that exhibited clinical or methodological homogeneity. Specifically, we conducted subgroup meta-analyses on the following:(1) MRI with a 3.0T field strength, (2) smoothed with an 8mm FWHM kernel. Due to the limited number of datasets available, no additional subgroup analyses were performed. The same statistical significance level as the primary analysis was applied. In addition, Cochran's Q statistic was used for heterogeneity testing to assess variability between studies. The *I*² statistic was also employed to calculate the percentage of overall variation attributed to heterogeneity. When the *P-*value of Cochran's Q is less than 0.05, it indicates significant heterogeneity between studies. The *I*² index ranges from 0% to 100%, with approximately 25%, 50%, and 75% representing low, moderate, and high heterogeneity, respectively(1). Finally, values were extracted from the relevant peaks, and Egger's test was used to explore the presence of publication bias in significant results (*P-*value < 0.05).

**Supplementary Results**

**Meta-regression analysis**

Our meta-regression analysis revealed a positive correlation between mean age and GMV changes in the following brain regions in AD patients: the bilateral precuneus (PCUN, MNI coordinates: x = 4, y = -54, z = 40; SDM-Z = 3.212, *P-*value = 0.005; 305 voxels), the left inferior parietal lobule (IPL, MNI coordinates: x = -50, y = -54, z = 38; SDM-Z = 3.337, *P-*value = 0.014; 60 voxels), and the right precentral gyrus (PreC, MNI coordinates: x = 42, y = 6, z = 34; SDM-Z = 3.518, *P-*value = 0.024; 17 voxels). The proportion of male gender showed a positive correlation with gray matter volume changes in the following brain regions: the left hippocampus/amygdala (HPC/AMYG, MNI coordinates: x = -20, y = -10, z = -16; SDM-Z = 2.436, *P-*value = 0.026; 69 voxels), as well as the left middle temporal occipital gyrus (TPOmid, MNI coordinates: x = -56, y = -10, z = -14; SDM-Z = 3.156, *P-*value = 0.013; 59 voxels). The results of the regression meta-analysis are presented in **Supplementary Table 5**.

**Subgroup meta-analysis**

In the subgroup analyses, the results for the subgroups with a 3.0T field strength and those smoothed with an 8mm FWHM kernel were largely consistent with the main analysis results. Specifically, significant GMV reductions in brain regions such as the right STG, MTG, and SMG were consistently observed across subgroups **(Supplementary Table 3-4**, **Supplementary Figure 1 )**. Overall, the primary findings were robust across different subgroups. However, subgroup sample size appeared to influence the statistical extent of some clusters, warranting caution in over-interpreting subgroup-specific differences.

**Reference**

1 Higgins JP, Thompson SG, Deeks JJ, Altman DG. Measuring inconsistency in meta-analyses. Bmj. 2003;327:557-60.

**Supplementary Figure**

**
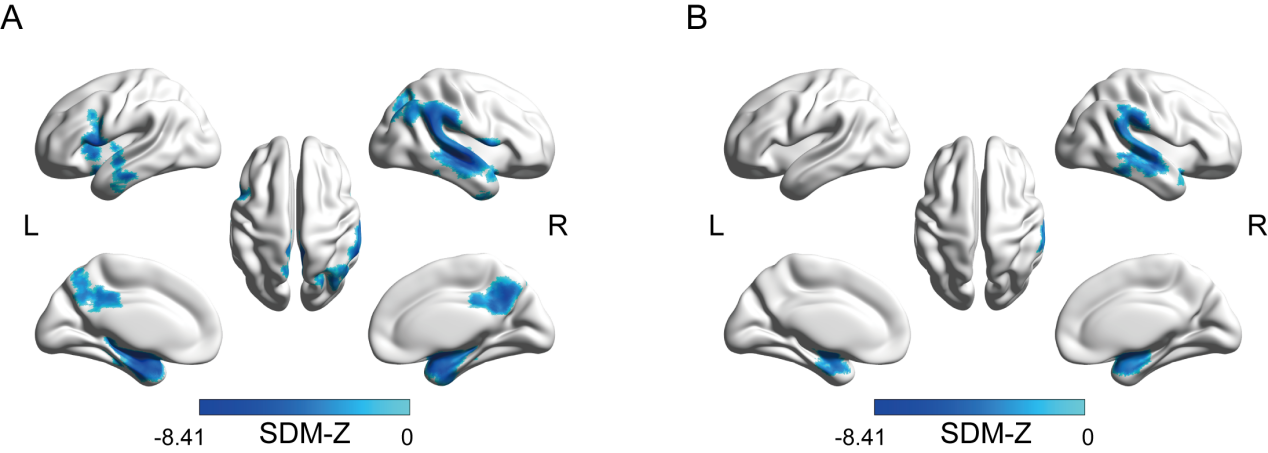
**

**Supplementary Figure 1. Gray matter volume changes in the subgroups.** **A.** Subgroup smoothed with an 8mm FWHM kernel. **B.** Subgroup with MRI using a 3.0T field strength.

**Abbreviations:** L, left; R, right; SDM, seed-based *d* mapping; FWHM, Full Width at Half Maximum; MRI, Magnetic Resonance Imaging.

**
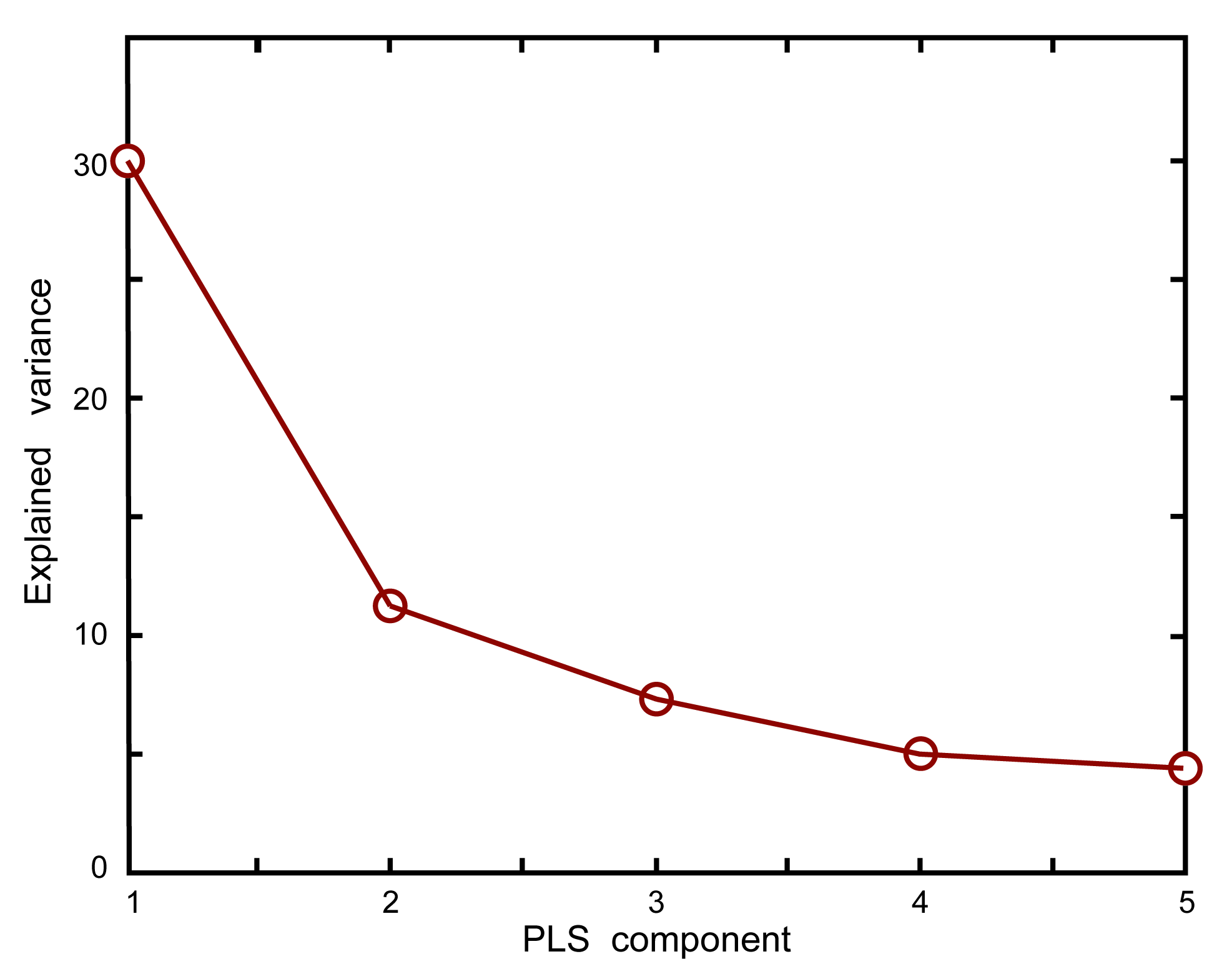
**

**Supplementary Figure 2. The variance explained by the top five components following PLS regression.** The first PLS component (PLS1) explained >20% of the variance.

**Abbreviations:** PLS, partial least squares.
